# Psychosis in Parkinson’s Disease: a Case Report of Diagnosis and Management

**DOI:** 10.1192/j.eurpsy.2023.2305

**Published:** 2023-07-19

**Authors:** R. P. L. Andrade, N. P. Gil, A. L. Costa, J. Brás, N. Castro, R. Sousa, R. P. Vaz, J. Martins, E. Almeida, J. Abreu, H. Afonso

**Affiliations:** Department of Psychiatry and Mental Health, Centro Hospitalar Tondela-Viseu, Viseu, Portugal

## Abstract

**Introduction:**

Psychosis is a frequent complication in patients diagnosed with Parkinson’s Disease (PD). Characterized mainly by visual hallucinations and paranoid delusions, it occurs most frequently, but not exclusively, as an adverse effect of antiparkinson medications. Nevertheless, cognitive impairment and dementia, as a frequent feature of PD, needs to be considered for differential diagnosis.

**Objectives:**

Our main objective is to report a case of PD Psychosis, its diagnosis and management and complement it with a non-systematic review of literature.

**Methods:**

Patient file consultation and an additional research, based on the key words “Psychosis” and “Parkinson’s Disease”, using Pubmed as database.

**Results:**

A 53-year-old female, diagnosed with Juvenile Parkinson’s Disease since age 45 and, as expected, polimedicated with antiparkinson medication. Without any relevant psychiatric background, she was admitted to the emergency department for disorganized behaviour, with 2 weeks of evolution. There, it was also possible to determine the presence of auditive hallucinations and persecutory delusions, associated with marked anguish.

After exclusion of any underlying cause for this symptomatology, inpatient treatment was proposed and accepted by the patient. In collaboration with the Neurology Department, a gradual reduction and optimization of antiparkinson drugs was conducted, associated with introduction of low doses of antipsychotic drugs, in this case Olanzapine. With this medication adjustments, clinical improvement was accomplished, with eventual fading and cessation of psychotic symptoms. Additionally, an irregularly intake of antiparkinson drugs was considered the most probably cause of this clinical decompensation.

**Conclusions:**

As present in literature, due to the chronicity and complexity of PD, stopping all antiparkinson drugs is not an option, even when psychotic symptoms, that could be a consequence of these drugs, are present. Therefore, a rigorous evaluation and management are mandatory, including the exclusion of other underlying causes and a careful therapeutic adjustment, with gradual reduction of antiparkinson drugs, addressing an eventual temporal relationship between the beginning of a specific drug and the onset of symptoms, and verification of therapeutic compliance, including an involuntary overdose. In cases of refractory symptoms, and after a risk-benefit assessment, pharmacologic treatment directed at these symptoms, low doses of anti-psychotics, may be necessary.

**Disclosure of Interest:**

None Declared

